# Forensic identification of severely degraded Atlantic salmon (*Salmo salar*) and rainbow trout (*Oncorhynchus mykiss*) tissues

**DOI:** 10.1186/2041-2223-1-12

**Published:** 2010-11-03

**Authors:** Sussie Dalvin, Kevin A Glover, Anne GE Sørvik, Bjørghild B Seliussen, John B Taggart

**Affiliations:** 1 Institute of Marine Research. P.O. Box 1870, Nordnes. N- 5817 Bergen, Norway; 2 Institute of Aquaculture, University of Stirling, Scotland, FK9 4LA, UK

## Abstract

**Background:**

Aquaculture is a globally important and rapidly growing industry. It contributes positively to the economy and sustainability of coastal communities, but it is not without regulatory challenges. These challenges are diverse, and may include identification of fish discarded in an illegal manner, biological discharge from fish ensilage tanks, and partially destroyed or processed tissues. Robust genetic tools are required by management authorities to address these challenges. In this paper, we describe nine species-specific primer sets amplifying very short DNA fragments within the mitochondrial DNA cytochrome c oxidase (COI) gene, which were designed to permit diagnostic identification of degraded DNA from two of the most commonly farmed salmonids in Europe and North America.

**Results:**

Of the nine designed primer sets, six were found to be species-specific (four Atlantic salmon, two rainbow trout), whereas the remaining three sets (two Atlantic salmon, one rainbow trout) also amplified a product from other, closely related, salmonid DNA templates. Screening of DNA templates from 11 other non-salmonid native fish species did not produce PCR products with any of the primer sets. Specific tests confirmed the ability of these markers to identify Atlantic salmon and rainbow trout tissues in treated food products, chemically treated ensilage waste and fillets left to degrade in saltwater for up to 31 days at 15°C. Importantly, these markers provided diagnostic identification in cases where other genetic methods failed because of degraded DNA quality.

**Conclusions:**

Results from this study demonstrate that amplification of very short DNA fragments using species-specific primers represents a robust and versatile method to create cheap and efficient genetic tests that can be implemented in a range of forensic applications. These markers will provide fishery, aquaculture and food regulatory authorities with a method to investigate and enforce regulations within these industries.

## Background

Aquaculture is playing an increasingly important role in meeting global protein requirements. Production of domesticated rainbow trout (*Oncorhynchus mykiss, Walbaum*) has a long history, and takes place in a large number of countries [1,2]. Aquaculture production of the Atlantic salmon (*Salmo salar *L.) was established in the 1960s, and in Europe this species represents the primary farmed fish in terms of both biomass and economic value. Norway has led this development, producing over 700,000 tons of Atlantic salmon and nearly 100,000 tons of rainbow trout in 2008 [3].

Aquaculture production of rainbow trout is based upon freshwater rearing or a mixture of freshwater and marine rearing, whereas production of Atlantic salmon almost exclusively involves a combination of juvenile rearing in freshwater and growing to market size in marine cages. Marine rainbow trout and Atlantic salmon farms tend to be located in sheltered coastal areas, and they contribute employment and financial opportunities to coastal communities. However, challenges remain concerning the mitigation of the negative effects of aquaculture, including genetic interactions [4,5], pathogens [6] and fish-farm effluent discharge [7]. Meeting such challenges involves, among other issues, the requirement to monitor the environmental effects and the ability to detect infringements of legislation. The latter often requires forensic investigation [8,9].

The application of DNA methods for identifying and authenticating fish and fishery products is increasing. These molecular tests [10,11] include a wide range of approaches of varying technical sophistication and cost, which exploit diagnostic polymorphisms within both mitochondrial (mt)DNA and nuclear (n)DNA genomes. Sequencing [12], restriction fragment length polymorphism [13] and species-specific PCR [14,15] approaches have been successfully implemented to identify partially degraded and otherwise compromised products. In general, mtDNA targeted methods have predominated in such studies, because of the general robustness and higher cellular copy number of mtDNA compared with nDNA [16].

One of the major challenges related to all forms of marine aquaculture is containment. In Norway, where fish farm escapees are recorded by the Directorate of Fisheries, the yearly total of farmed escapees were as high as 921,000 in 2006 for Atlantic salmon and 315,000 in 2007 for rainbow trout. [17,18]. Although DNA methods to identify the farm of origin have recently been developed and successfully implemented for both Atlantic salmon [8,19] and rainbow trout [20], there is a need to expand the repertoire of forensic tools to assist aquaculture and fishery agencies in regulation and law enforcement. For example, diagnostic identification of severely degraded tissues resulting from dead fish dumped illegally into the sea or of potential leakage of effluent from fish decomposition tanks located on farms represent two specific cases in which Norwegian enforcement agencies have requested technical assistance for forensic identification of biological matter.

To meet the needs of authorities enforcing law in aquaculture management, the aim of this study was to establish a rapid and cost-effective DNA-based method for the diagnostic identification of severely degraded Atlantic salmon and rainbow trout tissues. We hypothesised that amplification of very short mtDNA fragments would provide identification where other molecular methods would fail. Consequently, we designed species-specific primers that amplified very short mtDNA fragments within the cytochrome c oxidase (COI) gene [21,22]. Identifications using these markers were then compared in degradation tests with alternative molecular methods including mtDNA sequencing and fragment analysis of short tandem repeat nuclear loci.

## Results

### Species specificity of primer sets

**Figure 1:**
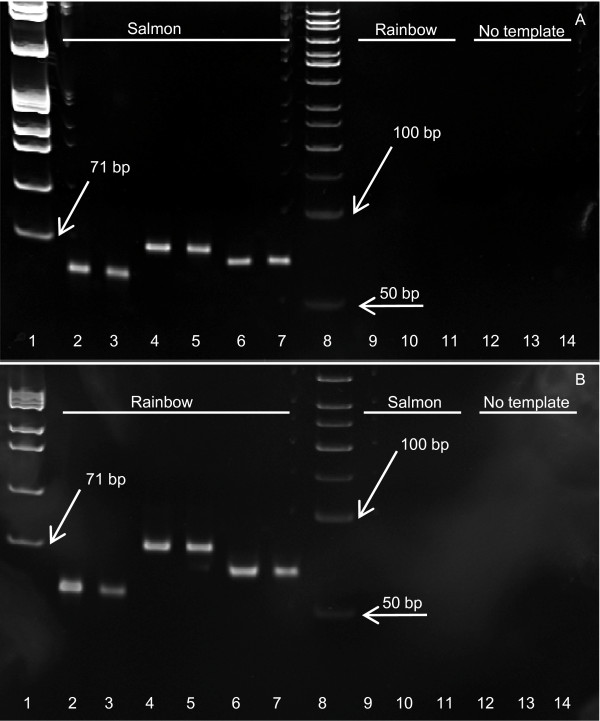
**PCR amplification of high-quality Atlantic salmon and rainbow trout DNA using species-specific short fragment cytochrome c oxidase (COI) primer sets**. Samples were separated by electrophoresis in 10% polyacrylamide gels. Template is indicated at the top of each gel. **(A) **DNA marker ϕX174/*Hae *III (lane 1), *SsaCOI030 *primers (lane 2-3), *SsaCOI208 *primers (lane 4-5), *SsaCOI334 *primers (lane 6-7), DNA marker 50 bp ladder (lane 8), *SsaCOI030 *primers (lane 9), *SsaCOI208 *primers (lane 10), *SsaCOI334 *primers (lane 11), *SsaCOI030 *primers (lane 12), *SsaCOI208 *primers (lane 13), *SsaCOI334 *primers (lane14). **(B) **DNA marker ϕX174/*Hae *III (lane 1), *OmyCOI032 *primers (lane 2-3), *OmyCOI203 *primers (lane 4-5), *OmyCOI340 *primers (lane 6-7), DNA marker 50 bp ladder (lane 8), *OmyCOI032 *primers (lane 9), *OmyCOI203 *primers (lane 10), *OmyCOI340 *primers(lane 11), *OmyCOI032 *primers (lane 12), *OmyCOI203 *primers (lane 13), *OmyCOI340 *primers (lane14).

The specificity and efficiency of the designed primer sets were initially characterised using high molecular weight DNA template obtained from fresh fish samples. In the first instance, six primer sets were examined (*OmyCOI032, 203 *and *340*, and *SsaCOI030, 208 *and *334*). In all cases, single clean PCR amplicons of expected size were produced for same species template, but no product was observed when using reciprocal DNA as template (Figure 1). The assay was robust to annealing temperature variation, with identical results being obtained when reactions were conducted with annealing temperatures of 57°C or 64°C (data not shown).

When amplification in other salmonid species was explored (Table 1), two of the three sets of rainbow trout PCR primers (*OmyCOI032 *and *203*) failed to resolve a product for any of the three species tested (brown trout, Arctic charr and whitefish). The third set (*OmyCOI340*) did resolve a PCR fragment of expected size with Arctic charr DNA. Only one Atlantic salmon PCR primer pair (*SsaCOI208*) proved to be species-specific. The two remaining sets (*SsaCOI030 *and *334*) produced a single clean amplicon of expected size with brown trout template DNA, but no product with either Arctic charr or whitefish DNA. To obtain more than one diagnostic marker for Atlantic salmon, an additional three primer sets were designed, this time with particular reference to a brown trout COI sequence. All these additional three primer sets (*SsaCOI076, 213 *and *401*) proved to be species-specific, yielding amplicons of expected size with Atlantic salmon DNA alone from the five salmonid species tested. As reported above for the other primers, similar performance was noted at both higher and lower annealing temperatures, except for amplification of rainbow trout DNA using the *SsaCOI076 *primers at 57°C.

**Table 1:** Overview of PCR amplification product in salmonids using the short fragment COI primer sets.

Primer set	Species				
	
	*Salmo salar*	*Oncorhynchus mykiss*	*Salmo trutta*	*Salvelinus alpinus*	*Coregonus laveratus*
*OmyCOI032*	-	+	-	-	-
*OmyCOI203*	-	+	-	-	-
*OmyCOI340*	-	+	-	+	-
*SsaCOI030*	+	-	+	-	-
*SsaCOI208*	+	-	-	-	-
*SsaCOI334*	+	-	+	-	-
*SsaCOI076*	+	-	-	-	-
*SsaCOI213*	+	-	-	-	-
*SsaCOI401*	+	-	-	-	-

+ = Amplification of a PCR product of appropriate size using the indicated DNA template; - = no product detected.

When tested against a panel of additional species DNAs (herring, redfish, tusk, cod, coalfish, pollock, plaice, monkfish, haddock, Atlantic halibut, mackerel), no specific PCR products were observed for any of the nine short fragment primer sets designed to salmonid COI genes.

### PCR amplification of decayed and processed DNA samples

**Figure 2:**
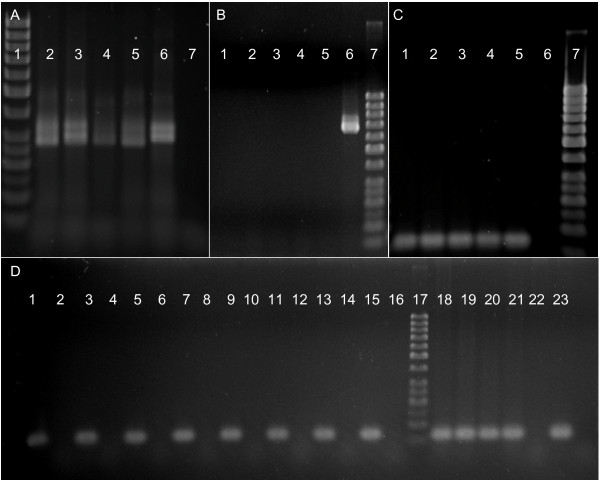
**PCR amplification of decayed and degraded DNA**. **(A) ***Ssp2201 *Atlantic salmon-specific microsatellite primers used on ensilage sample. Samples were separated by electrophoresis in 2% agarose gels. DNA marker 50 bp ladder (lane 1), ensilage samples (lane 2-5), high-quality genomic DNA extracted from fresh salmon (lane 6), no template (lane 7). **(B) **Fish primers used on canned products. Canned salmon fillet (lane 1-2), canned salmon pâté (lane 3-4), no template (lane 5), high-quality genomic DNA extracted from fresh salmon (lane 6), DNA marker 50 bp ladder (lane 7). **(C) ***SsaCOI030 *primers used on canned products. Canned salmon fillet (lane 1-2), canned salmon pâté (lane 3-4), high-quality genomic DNA extracted from fresh salmon (lane 5), no template (lane 6), DNA marker 50 bp ladder (lane 7). **(D) ***SsaCOI030 *primers used on seawater decayed Atlantic salmon and rainbow trout fillet and ensilage. Atlantic salmon fillet collected on days 3, 5, 7, 10, 13, 17, 21, 24, 28 and 31 (lane 1, 3, 5, 7, 9, 11, 13, 15), rainbow trout fillet sampled on the same days as Atlantic salmon (lane 2, 4, 6, 8, 10, 12, 14, 16), DNA marker 50 bp ladder (lane 17), ensilage (lane 18-21), no template (lane 22), high-quality genomic DNA extracted from fresh salmon (lane 23).

The ability of the designed primer sets to detect Atlantic salmon and rainbow trout mtDNA from degraded salmonid tissue samples compared with other existing markers (two microsatellites and the standard barcoding COI gene fragment), is summarised in Table 2. The microsatellite primers sets were the least successful in detecting expected DNA template. Apart from amplification in positive control samples (non-degraded DNA) the only product observed was from the ensilage DNA template amplified with *Sp2201 *primers. Even in this case, the amount of product was low and the result ambiguous in some samples (Figure 2A). Using barcoding COI primers, amplification from both Atlantic salmon pâté and canned products was unsuccessful (Figure 2B). Template DNA extracted from ensilage and from samples at all time-points within the tissue decay experiment did resolve several amplicons of approximately 700 bp, corresponding to expected size. However, subsequent cloning and sequencing of PCR products from the ensilage assay and two timepoints (3 and 31 days) from the decay experiment revealed multiple COI sequences. In addition to the expected Atlantic salmon sequence, BLAST analysis also identified sequences with closer homology to other species, of both prokaryotic and eukaryotic origins. By contrast, all short fragment COI primer sets designed in this study successfully amplified the species-specific fragments from the relevant degraded sample. This included samples from canned products (Figure 2C) and from all stages (3-31 days decay) of the fillet decay experiment (Figure 2D). In the degraded and canned samples, no crossamplification between salmon and rainbow trout samples or other unexpected amplification products were detected.

**Table 2:** Summary of PCR amplifications using a range of primers and DNA templates of varying quality.

Tissue source	Primer sets				
	Short fragment COI		Barcode	Microsatellite	
	*O. mykiss*-specific	*S. salar*-specific	*Fish1*	*Omm1303*	*Ssp2201*
Decayed fillets	+	+	-	-	-
Ensilage	NA	+	-	NA	[+]
Salmon pâté	NA	+	-	NA	-
Canned salmon fillet	NA	+	-	NA	-

COI = cytochrome c oxidase I

+ = Amplification of a PCR product of appropriate size using the indicated DNA template; - = no product detected; [+] = amount of PCR product was so low that interpretation of the result was ambiguous; NA = not applicable.

### PCR amplification of the unknown sea sample

PCR using primers specific to Atlantic salmon and rainbow trout revealed no amplification of products in any of the eight DNA isolates taken from the sea sample. Amplicons were generated using the barcoding COI primers in three of the eight DNA extracts but subsequent cloning and sequencing revealed multiple COI sequences having much greater similarity to other organisms, including brown seaweed (*Scytosiphon lomentaria*) and goldsinny wrasse *(Ctenolabrus rupestris)*. With the latter result indicating that amplifiable template DNA was present within the sea sample, it is unlikely that it originated from either Atlantic salmon or rainbow trout carcases.

## Discussion

Illegal, unreported and unregulated fishing represents a major threat to the sustainable exploitation of the world's marine resources [23]. Consequently, the application of molecular genetic tools in the management of fisheries, aquaculture and wildlife resources in general is gaining global importance. This study reports the successful development of diagnostic markers for Atlantic salmon and rainbow trout, which permitted the amplification of severely degraded DNA obtained from canned fish products, ensilage and heavily decayed fish tissue. These markers will provide management authorities with a tool to increase enforcement in a range of forensic applications.

The primers developed in this study were demonstrated to enable reliable species-specific identification under a range of amplification temperatures and on a range of degraded DNA templates likely to be available for forensic investigation. Although the species comparisons were not exhaustive, the fact that six of the primer sets did not amplify products from other high-quality salmonid template DNA indicates the likely overall robustness of the assays. However, it is not possible to exclude the possibility that false positive amplifications for individual primer sets may occur occasionally from DNA of species not assayed in the current study. It would be prudent, therefore, to assay multiple primer sets for identification purposes and to make a positive assignment only when data from all markers are consistent. The practicality of undertaking multiple PCR assays is aided by the fact that all nine primer sets share identical cycling parameters.

Examples of molecular genetics tools applied to fish and fisheries-related challenges are highly diverse, including competition fraud [24], identification of farm of origin of escapees, [8], illegal harvesting [25] and mixed stock fisheries [26]. Many of these applications relate to population identifications, which often exploit a combination of private and allele frequency differences observed in highly polymorphic markers, which are tested by a variety of statistical approaches [27]. However, although similar approaches have been used for species identification [28], identification of processed food products containing fish most often relies upon the analysis of diagnostic molecular markers not requiring statistical treatment [10,11]. With the increase in interest in fish barcoding [29], the availability of sequence data within the COI gene will provide the opportunity for further development of species-specific markers. Although it is acknowledged that sequencing of the COI gene can produce species identification in many cases, as reported here, species-specific amplification of short DNA fragments will provide an alternative viable and technologically simple approach for severely degraded tissues contaminated with other biological material. Similarly, PCR-based techniques exploiting amplification of short mtDNA has also been applied successfully to human forensics for identification of degraded samples [30].

There was no obvious qualitative difference in product yield from templates degraded in seawater from 3 to 31 days, or from ensilaged or processed foods. This is testament to the resilience of mtDNA to degradation, and also suggests that the primer sets could still prove to be informative for even more severely degraded material. The inability to detect Atlantic salmon or rainbow trout in the sea sample with the species-specific primers developed here indicates that the sea sample did not include significant amounts of DNA from either of these species. As this sample was taken in the vicinity of both rainbow trout and Atlantic salmon farms and an aquaculture fish processing factory, this result demonstrates that, unlike amplification with the barcoding primers and clone sequencing, these primer sets do not easily pick up false positives from sampling in the proximity of these operations or from other contaminating DNA present in the sample.

Use of the primer sets reported here can also be extended beyond forensic purposes. The brown trout is the closest relative to the Atlantic salmon, and these species occur sympatrically in the wild. Several studies have documented hybridisation between these two species, which has been identified by a variety of molecular techniques [31-34]. The fact that four of the Atlantic salmon specific markers developed here were able to distinguish between these two species means that these primers can be used to make a preliminary identification (as a pre-screening technique) of maternal contribution to a suspected hybrid using relatively simple technology before a more technologically demanding approach such as sequencing is undertaken.

## Conclusions

The present study was designed to establish a rapid and robust PCR-based assay to perform diagnostic identification of severely degraded Atlantic salmon and rainbow trout tissues. We hypothesised that amplification of very short mtDNA fragments using species-specific primers located in the COI gene would provide amplification where other molecular techniques would be inadequate. Both the species-specificity of these markers and importantly, their superiority in performing identifications on severely degraded DNA compared with alternative methods was demonstrated.

We conclude that these markers can be applied to a range of forensic applications, and will provide management authorities with a useful technique for regulation enforcement.

## Methods

### Study strategy and design of diagnostic PCR assays

The aim of this study was to establish a straightforward diagnostic PCR protocol for unambiguous identification of severely decomposed Atlantic salmon and rainbow trout tissues. This would preferably comprise simple amplification and gel visualisation steps without recourse to additional procedures such as sequencing or post PCR restriction analysis. An mtDNA-based assay was considered to be appropriate, with attention focused on the extensively characterised ~650 bp DNA barcoding fragment of the COI gene [35]. Primers were specifically designed to amplify very small fragments (*~*50-75 bp) within this region, to maximise the probability of amplifying severely fragmented template DNA. Potential species-specific primer sets were then tested for specificity and robustness on a range of common fish and on tissues from the target species, which had been subject to various treatments including degradation in salt water, ensilage and food processing.

Sequence data used in primer design comprised barcoding COI voucher sequences for Atlantic salmon (Genbank accession numbers: EF609449, EU524350, EU524353); rainbow trout (EF609420, EU524217, EU524220, EU524222) and brown trout (EU524354) plus additional barcode COI sequences (unpublished) that were generated at the Institute of Marine Research from 16 Atlantic salmon and 16 rainbow trout individuals sourced from a range of farms or locations across Norway. Consensus sequences were constructed from the multiple Atlantic salmon and rainbow trout sequences using SeqMan contig assembly software (Lasergene Inc., Madison, WI, USA), using the default assembly settings. These two consensus sequences were then aligned and inspected visually using BioEdit software [36] to locate potential species-specific primer sites (20-30 bp long sequences having 100% similarity within species reads, but substantial dissimilarity between species). Two species-specific panels of PCR primer pairs, with predicted annealing temperatures above 60°C, were then designed with the aid of PrimerSelect software (Lasergene Inc.). Each panel comprised three PCR primer sets for short amplicons (50-75 bp) from different regions of the COI gene (Table 3). Primer pairs were preferentially selected. Later, to confidently discriminate Atlantic salmon from congeneric brown trout, a further panel of three Atlantic salmon specific PCR primer sets were designed by comparing the Atlantic salmon consensus sequence with the single brown trout COI voucher sequence available on Genbank (Table 3). Primer and amplicon nomenclature comprised the initial letters from the species binomial (*Ssa *or *Omy*), the gene acronym (COI) and a three digit code indicating the 5" start position (in bases) of each amplicon relative to the voucher sequences.(for example, *SsaCOI208*, *OmyCOI032*)

**Table 3:** Overview of PCR primer sets used in this study

Primer name	Sequence 5" to 3"	**T**_**a**_, **°C**^**a**^	Amplicon size, bp
**Short fragment COI primers**			
*OmyCOI032F*	ATAGTAGGCACCGCCCTGAGTCTAC	62.0	52
*OmyCOI032R*	CCGGCTGGCTTAGTTCCGCC		
*OmyCOI203F*	CTAATAATCGGAGCCCCTGATATG	62.0	73
*OmyCOI203R*	AGGATGGAGGAAGGAGTCAGAAG		
*OmyCOI340F*	CCCTCTAGCCGGCAACCTC	62.0	62
*OmyCOI340R*	GAAGGGAGAAGATAGTTAAATCAACAGAG		
*SsaCOI030F*	GAATAGTCGGCACCGCCCTAAGTCTCT	62.0	57
*SsaCOI030R*	CGCCAGGCTGGCTGAGTTCTGCT		
*SsaCOI208F*	AATCGGGGCCCCCGACATA	62.0	71
*SsaCOI208R*	GAAAGGAGGGAGGGAGAAGTCAAAAA		
*SsaCOI334F*	CTACCCCCCTCTAGCAGGTAATCTT	62.0	65
*SsaCOI334R*	GGGAAAAAATAGTTAAGTCAACGGAA		
*SsaCOI076F*	CCAGCCTGGCGCCCTTCTG	62.0	63
*SsaCOI076R*	AAGGCATGGGCTGTAACAATTACGTT		
*SsaCOI213F*	GGGCCCCCGACATAGCAT	62.0	65
*SsaCOI213R*	AAAGGAGGGAGGAGAAGTCAAAAA		
*SsaCOI401F*	CATTTGGCTGGTATTTCTTCAATTCTT	62.0	75
*SsaCOI401R*	AGCTGGGGGTTTTATATTAATAATGGTT		
**Barcoding COI primers**^**b**^			
*Fish1F*	TCAACCAACCACAAAGACATTGGCAC	55.0	704
*Fish1R*	TAGACTTCTGGGTGGCCAAAGAATCA		
**Microsatellite primers**			
*Omm1303F*^c^	GGAAGGAAAGGCACTT	55.0	285 to 375^e^
*Omm1303R*^c^	TCTACACCAGGAGAGAGTAAT		
*Sp2201F*^d^	TTAGATGGTGGGATACTGGGAGGC	55.0	250 to 350^e^
*Sp2201R*^d^	CGGGAGCCCCATAACCCTACTAATAAC		

^a^T_a _= the routine PCR annealing temperature used for each primer set;

^b^universal barcoding primers [37]; ^c^rainbow trout microsatellite [39]; ^d^Atlantic salmon microsatellite [38]; ^e^microsatellite allele sizes were derived from farmed fish surveys.

Three additional primer sets were used as comparators for successful PCR amplification from rainbow trout and Atlantic salmon DNA samples. These were a standard COI barcoding primer set (*Fish 1 *[37]) that works effectively for salmonids and two microsatellite markers, Atlantic salmon specific *Sp2201 *[38] and rainbow trout specific *OMM1303 *[39], which are routinely used in the Institute of Marine Research laboratory. Details of these primers, including expected amplicon sizes, are given in Table 3.

### Samples and DNA extraction

Canned Atlantic salmon fillet and pâté (Stabburet, Norway) and frozen Atlantic salmon and rainbow trout fillets, complete with skin, were purchased in a local supermarket. To test the ability of the primers to amplify degraded tissues, the frozen Atlantic salmon and rainbow trout fillets were kept in fish tanks containing running seawater at 15°C. Samples of these fillets were then taken at 3, 5, 7, 10, 13, 17, 21, 24, 28 and 31 days after placing them into the tanks. A sample of ensilage waste of Atlantic salmon was collected from an experimental research farm (Matre Field Station, Matre, Norway). This ensilage tank contained remains of dead Atlantic salmon mixed with sodium hydroxide. Such tanks are commonplace on commercial farms for temporary storage of dead fish before transport to factories for processing.

To test the specificity of the designed primers, a range of native fish species were also examined. Most of these were purchased from a local fish retailer, and comprised herring (*Clupea harengus*), redfish (*Sebastes marinus*), tusk (*Brosme brosme*), cod (*Gadus morhua*), coalfish (*Pollachius virens*), pollock (*Pollachius pollachius*), plaice (*Pleuronectes platessa*), monkfish (*Lophius piscatorius*), haddock (*Melanogrammus aeglefinus*), Atlantic halibut (*Hippoglossus hippoglossus*) and mackerel (*Scomber scombrus*). Samples of DNA from three other salmonid species (brown trout (*Salmo trutta*), Arctic charr (*Salvelinus alpinus*) and whitefish (*Coregonus laveratus*)) were available from laboratory archives.

Finally, a 2 litre sample of unidentified floating biological material mixed with sea water was collected from an undisclosed coastal location in Norway. This sample, herein referred to as the 'sea sample', was taken in the summer of 2008 in the vicinity (<1 km) of a commercial Atlantic salmon farm and a factory that processes fish waste from aquaculture installations. The legal authorities were interested as to whether the sample could be related to biological discharge from either nearby operation.

DNA was extracted from fish fins or from a mixture of skin and muscle from the rainbow trout and Atlantic salmon samples that had been exposed to a variety of treatments (described above). For the sea sample, DNA was extracted from a homogenised mixture of the seawater and floating biological matter sample. For all samples, DNA was extracted using a commercially available column purification protocol (DNAeasy Kit, Qiagen, Valencia, CA, USA). DNA was quantified spectrophotometrically (Nanodrop ND-1000; Thermo Scientific, Wilmington, DE, USA). Except for tissues exposed to degradation, DNA was extracted from a minimum of two individuals in separate isolations. For the canned food samples (potentially containing tissue from multiple individuals) two separate DNA extractions from each can were performed. Eight separate DNA extractions were performed on the homogenised sea sample mixture.

### PCR screening

Each short fragment COI gene PCR reaction (25 μL total volume) contained ~25 ng DNA, 5 μL GoTaq^® ^buffer, 2.5 mM MgCl_2_, 200 μM each dNTP, 0.25 μM each primer and 0.15 U *Taq *polymerase (GoTaq^®^, Promega Corp., Fitchburg, WI, USA). DNA was amplified in a thermal cycler (Mastercycler epgradient S; Eppendorf, Hamburg, Germany) using the following cycling conditions: initial denaturation at 94°C for 2 min, followed by 30 cycles of denaturation at 94°C for 30 seconds, 62°C for 30 seconds and extension at 72° for 30 seconds, then a final extension at 72°C for 10 minutes. To examine the robustness of this temperature profile for each assay, PCR reactions were then repeated with annealing temperatures 2°C above and 5°C below the selected optimum. Positive and negative controls (no template) were used throughout. The *Fish1*, the *Ssp2201 *and the *Omm1303 *primer sets used the same conditions, but with an annealing temperature of 55°C.

### Sequencing

Where amplicon verification was required, fragments were either cloned (TOPO TA Cloning Kit; Invitrogen Corp., Carlsbad, CA, USA) before sequencing, or purified (ExoSAP-IT; Affymetrix-USB, Cleveland, OH, USA) and sequenced directly on an automated sequencer (ABI 3700 with BigDye 3.1 sequencing reagents; Applied Biosystems, Foster City, CA, USA).

## Competing interests

The authors declare that they have no competing interests.

## Authors' contributions

SD planned and performed PCR, cloning and sequencing reactions, and wrote the paper together with KAG and JBT. KAG planned and supervised the study, and wrote the paper together with SD and JBT. AGES and BBS performed extraction of genomic DNA, PCR and sequencing work. JBT supervised the study, and wrote the paper together with SD and KAG. All authors read and approved the manuscript.
